# Prevalence and risk factors of overweight and obesity in adolescents in Malang, East Java-Indonesia

**DOI:** 10.1186/1687-9856-2013-S1-O50

**Published:** 2013-10-03

**Authors:** Ariani Harijono, Anik Puryatni, Haryudi Adji Cahyono, Mardhani Yosoprawoto

**Affiliations:** 1 Child Health Department Saiful Anwar General Hospital/Medical Faculty Brawijaya University, Indonesia

## 

Obesity and overweight in adolescents has more than tripled in the past 30 years. They are among the easiest medical conditions to recognize but most difficult to treat and has both immediate and long-term effects on health and well-being. We are interested in determine the prevalence of overweight and obesity in adolescents and to identify the risk factors.

A cross sectional study was carried out on 346 adolescents of senior high school aged from 15-18 years old in Malang City, Indonesia. Anthropometric measurements included body weight, height, and mid-upper arm circumference (MUAC). The nutritional status was classified based on Body Mass Index (BMI) using the WHO standard criteria. Dietary recalls were collected to assess the quality of food. A systematic random sampling was made according to school grade. Most subjects (72.8%) had normal weight, 11.8% were underweight, 7.2% were overweight and 8.1% were obese. Among overweight group, 60% were boys, while among obese group, 50% were boys. On multivariate regression analysis, it was found that energy expenditure had significant negative correlation with the occurrence of overweight and obesity (p <0.001) while energy intake and lifestyle had a significant positive correlation (p <0.001 and p <0.001). Energy intake and lifestyle were proportionally related to overweight and obesity.

